# A New Approach to Diagnose Parkinson's Disease Using a Structural Cooccurrence Matrix for a Similarity Analysis

**DOI:** 10.1155/2018/7613282

**Published:** 2018-04-24

**Authors:** João W. M. de Souza, Shara S. A. Alves, Elizângela de S. Rebouças, Jefferson S. Almeida, Pedro P. Rebouças Filho

**Affiliations:** Laboratório de Processamento de Imagens, Sinais e Computação Aplicada (LAPISCO), Instituto Federal de Educação, Ciência e Tecnologia do Ceará, Fortaleza, CE, Brazil

## Abstract

Parkinson's disease affects millions of people around the world and consequently various approaches have emerged to help diagnose this disease, among which we can highlight handwriting exams. Extracting features from handwriting exams is an important contribution of the computational field for the diagnosis of this disease. In this paper, we propose an approach that measures the similarity between the exam template and the handwritten trace of the patient following the exam template. This similarity was measured using the Structural Cooccurrence Matrix to calculate how close the handwritten trace of the patient is to the exam template. The proposed approach was evaluated using various exam templates and the handwritten traces of the patient. Each of these variations was used together with the Naïve Bayes, OPF, and SVM classifiers. In conclusion the proposed approach was proven to be better than the existing methods found in the literature and is therefore a promising tool for the diagnosis of Parkinson's disease.

## 1. Introduction

According to the World Health Organization, neurological disorders such as Parkinson's disease, multiple sclerosis, Alzheimer's disease, epilepsy, shingles, and stroke are nervous system diseases that affect the brain, the spine, and the nerves that connect them. Approximately 16 in 60 people suffer from some neurological disease [1]. Parkinson's disease (PD), first described by Parkinson [2], is a degenerative disease of the central nervous system associated with a chronic and progressive movement disorder [1]. Parkinson's Disease Foundation claims that this disease affects about 7–10 million people worldwide and 4% of people with PD are diagnosed before the age of 50. The cause is unknown and there is no cure for PD, but an early diagnosis helps in the treatment that continues throughout the patient's life.

PD studies, in the computational field, are mainly focused on diagnosing the disease. The literature shows that some works aim to recognize the presence or absence of PD and identify the patients degree of severity [3, 4], and another extracts features from handwriting exams [5], among others [6–12]. Most of the studies use signals from exams to make a diagnosis. However, studies related to a diagnosis through handwriting exams (handwriting exams based on the quality of the patient's tracing results can be used for PD diagnosis) are quite scarce [5].

Handwriting exams may be conducted on paper [13] or by using more sophisticated methods such as digitizers [5] or even a smartphone [14]. This type of exam has advantages as it is easily obtainable and can also provide diversity, such as spirals, ellipses, connected syllables, connected words, and many other ways to test a patient's ability to trace such forms [15–19]. However, the extraction of the features is complicated since the paper exams have some printing error and the information in this type of exam is not so clear.

This paper compares handwriting templates and patients handwriting using a novel Structural Cooccurrence Matrix-based approach which relies on similarity metrics as attributes. This approach was used because feature extraction through cooccurrence between similar images appeared as a promising method for this application. The proposal was evaluated using three classifiers and the results were compared against those in [13].

The rest of this paper is organized as follows. The essentials of handwriting exams and some machine learning methods are explained in Sections 2 and 3, respectively.  Section 4 describes the Structural Cooccurrence Matrix features. Our proposal is presented in Section 5. The experimental setup is presented in Section 6. Then, the results and discussion are given in Section 7 and finally the conclusions are given in Section 8.

## 2. Diagnosis of Parkinson's Disease through Handwriting Exams

There are several examples in the literature that apply handwriting exams to diagnose PD. Drotár et al. recorded the examination time as a parameter for PD diagnosis [5] while Surangsrirat used a polar coordinates interpretation to define the features [20]. There are also works based on the difference between the patient's trace and the template [13].

Pereira et al. [13] introduced a new method to obtain the exam which is performed on paper and relies on underlining the template correctly. Pereira et al. also proposed a set of images composed of handwriting exams known as HandPD dataset.

**Figure 1:**
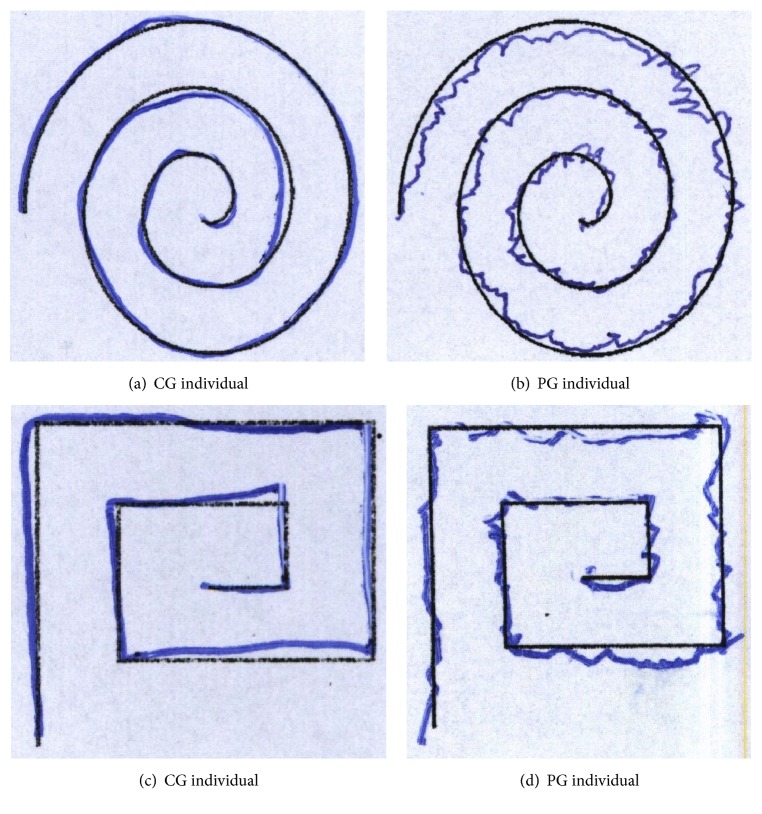
(a-b) Handwriting exams in a spiral format; (c-d) handwriting exams in a meander format [13].

The HandPD dataset [13] consists of 736 images from handwriting exams divided into two groups: the Control Group (CG) containing 144 images and the Patient Group (PG) containing 592 images. The exams were obtained from 92 individuals, in which 18 were healthy individuals (CG) and 74 were patients (PG) diagnosed with PD. These exams were performed at the Botucatu Medical School of the State University of São Paulo, Brazil, and include spiral and meander handwriting exam templates.  Figure 1 shows some samples from this dataset [13].

Pereira et al. [13] used an approach to define the dataset attributes based on the differences between the exam template (ET) and the handwritten trace (HT). Pereira et al. [13] described the HandPD dataset exams using the 9 attributes listed below:

(1) Root mean square (RMS) of the difference between HT and ET radius:
  (1) $$\begin{array}{c} RMS=\sqrt{\frac{1}{n}\overset{n}{\underset{i=0}{\sum }}{\left(r_{HT}^{i}-r_{ET}^{i}\right)}^{2}}, \end{array}$$
where *n* is the number of sample points drawn for each HT and ET skeleton and *r*_HT_^*i*^ and *r*_ET_^*i*^ denote the HT and ET radius, which is basically the length of the straight line that connects the *i*th sampled point, respectively, to the center of the spiral or meander.
(2) Maximum difference between HT and ET radius:
  (2) $$\begin{array}{c} \Delta_{max}=\underset{i}{argmax}\left\{\;\left|\;r_{HT}^{i}-r_{ET}^{i}\;\right|\;\right\}. \end{array}$$
(3) Minimum difference between HT and ET radius:
  (3) $$\begin{array}{c} \Delta_{min}=\underset{i}{argmin}\left\{\;\left|\;r_{HT}^{i}-r_{ET}^{i}\;\right|\;\right\}. \end{array}$$
(4) Another attribute is standard deviation of the difference between HT and ET radius.
(5) Mean relative tremor (MRT) [13] is a quantitative evaluation to measure the “amount of tremor” of a given individual's HT:
  (4) $$\begin{array}{c} MRT=\frac{1}{n-d}\overset{n}{\underset{i=d}{\sum }}\left\{\;\left|\;r_{ET}^{i}-r_{ET}^{i-d+1}\;\right|\;\right\}, \end{array}$$
where *d* is the displacement of the sample points used to compute the radius difference.
(6) There is maximum ET radius.
(7) Another attribute is minimum ET radius.
(8) There is also standard deviation of HT radius.
(9) The last one is the number of times the difference between HT and ET radius changes from negative to positive or the opposite.

## 3. Overview of Machine Learning Methods

Pereira et al. [13] evaluated their approach using experiments which involved the Naïve Bayes, Optimum-Path Forest (OPF), and Support Vector Machines (SVM) classifiers. Therefore, we conducted our experiments with the same aforementioned classifiers in order to compare our proposal with Pereira et al.'s.

All three classifiers deal with recognition problems in different ways. Based on Bayes' Theorem, the Naïve Bayes classifier is a probabilistic approach that makes a strong independence assumption among the predictors [21]. The common terms in such a theorem, a priori and a posteriori, are related to an indication of known probabilities and the probability in future indications, respectively.

On the other hand, the OPF classifier designs the recognition problem based on the Graph Theory in a particular feature space [22]. A competition process is established among some patterns, called prototypes that are determined during the training step, and each connected pattern carries its cost. After that, the optimal-path forest is computed using the Image Forest Transform (ITF) algorithm. The LibOPF library [23] was used to implement the OPF classifier used in this work.

The SVM classifier is based on the Vapnik statistical learning theory [24]. The main goal of this classifier is to find an optimal hyperplane able to separate the patterns of each label. This optimal hyperplane is obtained through the linear separation of the patterns in space, and after the feature space is defined the SVM determines the optimal hyperplane.

## 4. Structural Cooccurrence Matrix (SCM)

In this section, we present a brief description of the SCM, which is the basis for the approach of this article, as proposed by Bezerra Ramalho et al. [25].

**Figure 2:**
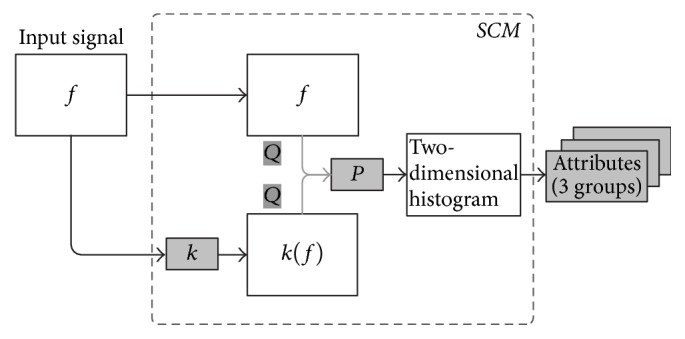
An example of an SCM [25].

Bezerra Ramalho et al. [25] introduced a general purpose structural image analytical method based on cooccurrence statistics saved in a matrix, namely, Structural Cooccurrence Matrix (SCM). This method analyzes, in an *n*-dimensional space, the relation between low-level structures of two discrete signals.  Figure 2 shows an example of this method.

This method has a variable *k* which is any invariant image filter that exposes saliences of the image *f*. Here, *k* is configurable for each application. Thus, depending on the a priori knowledge of the characteristics of the image being analyzed, *k* can be either a high-pass filter or a low-pass filter [25].

## 5. Proposed Approach

**Figure 3:**
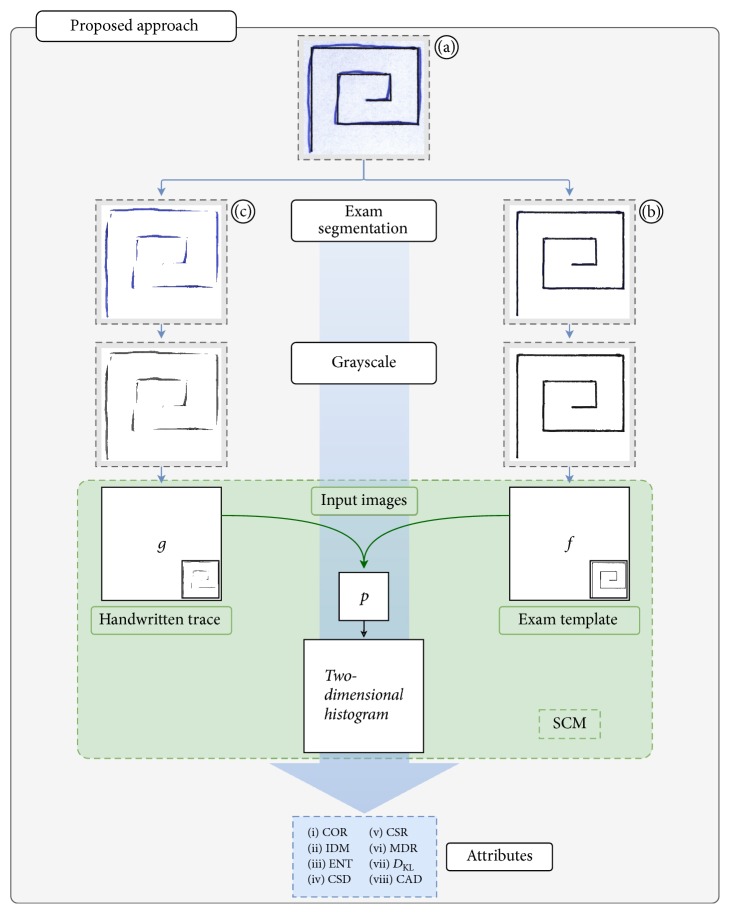
Flowchart of the proposed approach.

In this section, we present our SCM-based approach to diagnose PD. This new approach extracts features from the spiral and meander handwriting exams of the HandPD dataset [13].  Figure 3 shows the proposed flowchart. This flowchart presents one of the combinations which uses handwritten trace (c) and exam template (b).

**Figure 4:**
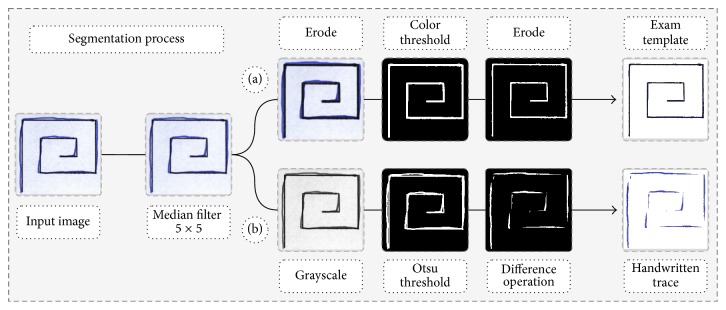
An example of the segmentation process: (a) segmentation of exam template; (b) segmentation of handwritten trace.

The first step is the exam segmentation which returns two new images: the exam template (ET) and the handwritten trace (HT). This step is presented in detail in Figure 4. The images are obtained by applying digital image processing techniques on the handwriting exams.

In order to obtain the ET segmentation the image is smoothed through a Median filter (5 × 5) to eliminate the noise picked up during the acquisition of the exam. Then, an erosion (9 × 9 ellipse structure) is applied to ensure that there is no discontinuity in the ET segmentation. After that, an empirically defined threshold is used. Finally, an erosion is applied again with the same structuring element to obtain the real size of the ET as shown in Figure 4.

In order to obtain the HT segmentation the image is also smoothed through the Median filter (5 × 5) to eliminate the noise picked up during the exams. Then, the handwriting exam is converted to the grayscale, after which the Otsu threshold is applied. Finally, we apply a difference operation between the grayscale image and the ET. These steps are shown in Figure 4.

The second step in our proposal converts the segmented exams to the grayscale for the next step. The third step is feature extraction from the images obtained after the segmentation and conversion to the grayscale. These images are used as the input to SCM as showed in Figure 2. The feature extraction through the SCM is a method to analyze the relationship between signals, in this case, in a two-dimensional space.

A slight modification was made to the original SCM method. The SCM has two input parameters, an image and a filter. We replaced the filter with another image. The new configuration of the input parameters is presented as an example in Figure 3. This proposal is intended to enhance the differences between the two input images by computing the similarity approximation between the patient's trace and the exam template. There is no need to configure a filter as in this SCM method the filter was replaced for another image.

Three combinations of three images were proposed as the SCM inputs, which were then used for a complete analysis: (i) handwriting exam and handwritten trace; (ii) handwriting exam and exam template; and (iii) handwritten trace and exam template.

The scalar attributes, obtained through the SCM method, are computed by the SCM generated between the input images. These attributes are divided into three groups: statistical group, information group, and divergent group [25]. All these attributes are computed based on the SCM matrix represented by **M** = **m**_*ij*_, and some are related to the marginal distribution **P** of the SCM. A brief description of each attribute is given below:

(i) Correlation (COR) measures how the information is correlated. The COR is given by
  (5) $$\begin{array}{c} COR=\overset{N-1}{\underset{ij=0}{\sum }}m_{ij}\frac{\left(i-\mu_{i}\right)\left(j-\mu_{j}\right)}{\sqrt{\sigma_{i}2\sigma_{j}2}}\in \left[\;-1,1\;\right], \end{array}$$
where *μ*_*i*_ and *μ*_*j*_ are the average value of rows and columns of** M**. *σ*_*i*_ and *σ*_*j*_ are the standard deviation of rows and columns of** M**.
(ii) Inverse difference moment (IDM) measures the homogeneity and it is given by
  (6) $$\begin{array}{c} IDM=\overset{N-1}{\underset{ij=0}{\sum }}\frac{m_{ij}}{1+\left(\left|i-j\right|\right)}\in \left[\;0,1\;\right]. \end{array}$$
(iii) Entropy (ENT): the randomness of the information is measured and it is given by
  (7) $$\begin{array}{c} ENT=-\overset{N-1}{\underset{ij=0}{\sum }}m_{ij}\log\left(\;m_{ij}\;\right). \end{array}$$
(iv) Chi-square distance (CSD): since the **P**_*i*_^0^ = **m**_*ij*_ = *j* and **P**_*i*_^*e*^ = ∑_*j*=0_^*N*−1^**m**_*ij*_ > = *i*,
  (8) $$\begin{array}{c} CSD=\overset{N-1}{\underset{i=0}{\sum }}\frac{{\left(P_{i}^{0}-P_{i}^{e}\right)}^{2}}{P_{i}^{e}}. \end{array}$$
(v) Chi-square distance ratio (CSR): with the distributions of the quadrants I, **P**_*i*_^I^ = ∑_*i*=0_^*N*/2−1^∑_*j*=0_^*N*/2−1^**m**_*ij*_, and III, **P**_*i*_^III^ = ∑_*i*=*N*/2_^*N*−1^∑_*j*=*N*/2_^*N*−1^**m**_*ij*_, the CSR is given by
  (9) $$\begin{array}{c} CSR=\overset{N-1}{\underset{i=0}{\sum }}\frac{{\left(P_{i}^{I}-P_{i}^{m}\right)}^{2}}{P_{i}^{m}}, \end{array}$$
where **P**_*i*_^*m*^ = (**P**_*i*_^I^ + **P**_*i*_^III^)/2.
(vi) Mean absolute difference ratio (MDR) calculates the statistical dispersion, where MD_min_ = min(MD_*p*^*c*^_, MD_*p*^*r*^_), MD_max_ = max(MD_*p*^*c*^_, MD_*p*^*r*^_), and MD_*p*_ = ∑_*i*=0_^*N*−1^∑_*j*=0_^*N*−1^**P**(*S*_*i*_)**P**(*S*_*j*_) | *S*_*i*_ − *S*_*j*_|. The MDR is given by
  (10) $$\begin{array}{c} MDR=\frac{MD_{min}}{MD_{max}}, \end{array}$$
where *S*_*i*_, *S*_*j*_, *i* = 0,…, *N* − 1 are the indexes of the nonzero values of the marginal distribution **P**.
(vii) Divergence of Kullback Leibler (*D*_KL_) measures the information gain between bordering distributions and it is given by
  (11) $$\begin{array}{c} D_{KL}=\overset{N-1}{\underset{i=0}{\sum }}\log\left(\;\frac{P_{i}^{c}}{P_{i}^{r}}\;\right)P_{i}^{c}, \end{array}$$
  where **P**_*i*_^*c*^/**P**_*i*_^*r*^ = 0, where **P**_*i*_^*r*^ = 0.
(viii) Complementary absolute difference (CAD) compares the two probability distributions **P**_*i*_^*c*^ and **P**_*i*_^*r*^. The CAD is given by
  (12) $$\begin{array}{c} CAD=1-\overset{N-1}{\underset{i=0}{\sum }}\left|\;P_{i}^{c}-P_{i}^{r}\;\right|. \end{array}$$

At the end of the SCM process we have the attributes to be applied as pattern recognition inputs to any machine learning method.

## 6. Experimental Setup

We conducted some experiments with the handwriting exams used by Pereira et al. [13]. These exams concern the patient's ability to underline the two exam templates: spiral and meander. First, the spiral and meander exam attributes were extracted by the proposed approach presented in Section 5. Then, we applied three machine learning methods: Naïve Bayes, OPF, and SVM in 3 different experiments: (i) considering only the spiral exam attributes; (ii) considering only the meander exam attributes; and (iii) considering spiral and meander exam attributes together. That is, we evaluate the proposed approach using the two handwriting formats separately and also together. After that, we compared the results with those of Pereira et al. [13].

The number of HandPD dataset samples, used in all of the experiments, are divided into 368 spiral exam samples and 368 meander exam samples, resulting in 736 samples. We emphasize that each subset is divided into 296 samples from the CG and 72 samples from the PG.

All the three experiments were conducted with 75% for the training set, 25% for testing set, and the remaining 25% for the validation set. We applied a cross-validation with 20 runs for the reliability of the results. The setup of the OPF classifier was configured with the Euclidean distance and the SVM with the radial basis function. The SVM and OPF classifiers were automatically optimised by choosing the optimal parameters from each method. Parameters are considered optimal when the cross-validation estimates the minimal error [26–32].

Accuracy was used to classify the machine learning methods. This metric is defined using the terms obtained in the confusion matrix generated after applying the machine learning methods and refers to the closeness of a measured value to a standard or known value.

## 7. Results and Discussion

In this section, we provide an analysis of the results using the handwriting exams from Pereira et al. [13]. These exams present the patient's ability to underline the exam template. The results were obtained by applying machine learning methods to the features extracted from cooccurrence matrix obtained over the SCM extractor used in this work.

Table 1 presents the classification results of the combinations between the images obtained in the segmentation (ET and HT) and the handwritten exam using SCM, where the meander and spiral formats were evaluated separately and together. The handwritten exam, exam template, and handwritten trace are represented in Table 1 as** a**,** b**, and** c**, respectively.

Table 1 also shows that the combination of the meander and spiral exam obtained the lowest accuracy rates. The very significant difference between the spiral and meander formats justifies these low rates.

Another important factor, observed in the best results obtained with the classifiers in Table 1, was the predominance of the best results using the combination of the handwritten trace and the exam template which proves that these two are the most similar.

The results of this work were compared with the results obtained by Pereira et al. approach [13] in Table 2 which shows each classifier with subdivisions for the experiments using handwriting exams in the formats of meanders, spirals, and the combination of the meanders and spirals. Immediately after that, there is a new subdivision between the results obtained with our proposal and those of Pereira et al. [13]. In Table 2 the highest accuracy results are in underline font and the lowest ones in italic font, for each of the classifiers. Also, highlight in bold are the highest and the lowest accuracy results of both approaches and experiments.

The results obtained in this paper proved to be superior in all of the experiments in comparison to those of Pereira proposal [13]. The highest accuracy was 85.54% using the approach proposed. This highest result was provided with the SVM classifier, using the handwriting in a spiral format with the combination of the handwritten trace and the exam template. The lowest accuracy among the best results presented in Table 2 was 45.79%.

## 8. Conclusion

The proposal of this paper is based on the patient's trace and the exam template using the SCM method for a similarity approximation. The SVM classifier with the RBF kernel and handwriting in the spiral format proved being the most promising for this application. This configuration had an accuracy of 85.54% for the combination of the handwritten trace and the exam template in the feature extraction. The results obtained in this paper were 21.31% superior to the best result achieved by Pereira et al. approach [13].

We conclude that this is promising approach to help in the diagnosis of PD. Another advantage of this proposal is that there is no need to configure a filter to obtain the structured cooccurrence matrix, facilitating its application.

These results encourage us to propose future works for handwriting feature extraction.

## Figures and Tables

**Table 1:** Results from the best classifiers and combinations.

	Meander	Spiral	M/S
	Acc (%)	Acc (%)	Acc (%)
Bayes			
**a**⇔**c**	72.77 ± 9.91	69.57 ± 11.07	65.95 ± 2.05
**a**⇔**b**	75.11 ± 3.99	76.36 ± 4.26	68.38 ± 2.18
**c**⇔**b**	**77.72 ± 4.27**	**82.01 ± 5.53**	**71.39 ± 2.39**
OPF			
**a**⇔**c**	75.54 ± 3.76	69.73 ± 3.63	64.28 ± 2.05
**a**⇔**b**	68.59 ± 3.76	70.49 ± 3.63	61.13 ± 2.57
**c**⇔**b**	**77.50 ± 2.75**	**75.71 ± 4.39**	**67.21 ± 1.98**
SVM			
**a**⇔**c**	80.05 ± 1.80	78.04 ± 2.74	67.63 ± 2.73
**a**⇔**b**	75.00 ± 2.90	78.04 ± 2.85	65.30 ± 2.92
**c**⇔**b**	**82.23 ± 3.02**	**85.54 ± 3.62**	**74.13 ± 2.27**

**Table 2:** Comparison between the best results of this paper and the best results of the Pereira et al. approach [13].

Classif.	Database	Feature extractor	Accuracy (%)
*Bayes *	*Meander*	Pereira et al. approach [13]	*59.20 ± 4.78*
		*Proposed approach*	77.72 ± 4.27
	*Spiral*	Pereira et al. approach [13]	*64.23 ± 7.11*
		*Proposed approach*	82.01 ± 5.53
	*M/S*	Pereira et al. approach [13]	***45.79*** * ± * ***4.15***
		*Proposed approach*	71.39 ± 2.39

*OPF *	*Meander*	Pereira et al. approach [13]	*57.54 ± 6.35*
		*Proposed approach*	77.50 ± 2.75
	*Spiral*	Pereira et al. approach [13]	*52.48 ± 5.32*
		*Proposed approach*	75.71 ± 4.39
	*M/S*	Pereira et al. approach [13]	*55.86 ± 3.63*
		*Proposed approach*	67.21 ± 1.98

*SVM *	*Meander*	Pereira et al. approach [13]	*66.72 ± 5.33*
		*Proposed approach*	82.23 ± 3.02
	*Spiral*	Pereira et al. approach [13]	*50.16 ± 1.71*
		*Proposed approach*	**85.54** ± **3.62**
	*M/S*	Pereira et al. approach [13]	*58.61 ± 2.84*
		*Proposed approach*	74.13 ± 2.27
